# Supplementary material for: Kinesin-1 conformational dynamics are controlled by a cargo-sensitive TPR switch
Source: eLife. 2026 Apr 14;14:RP109462. doi: 10.7554/eLife.109462 (PMC13078783; doi:10.7554/eLife.109462)

WT WT-KinTag ElbowLock

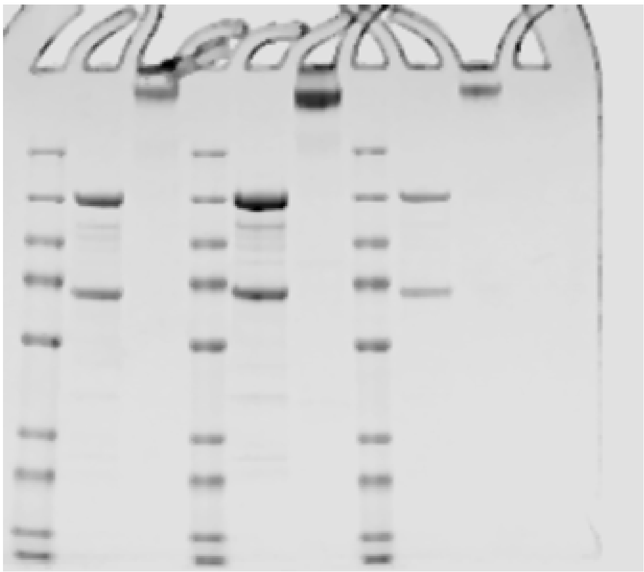

ElbowLock-Kintag ElbowLock-deltaTDS Not used

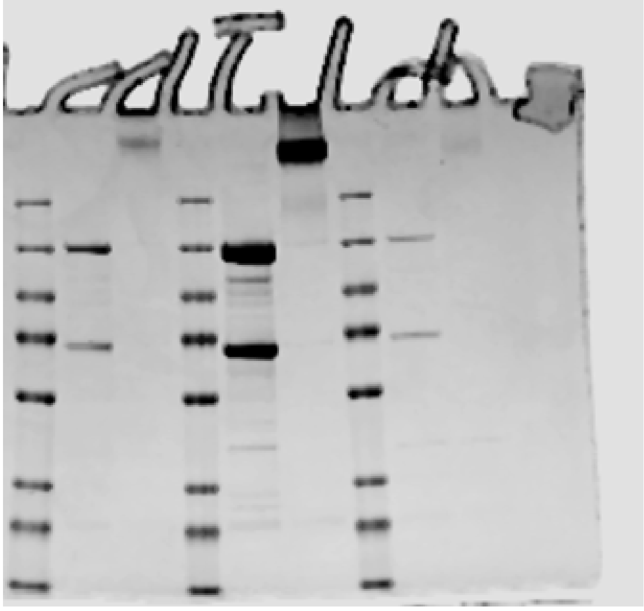

ElbowLock-Nanobody (bottom)

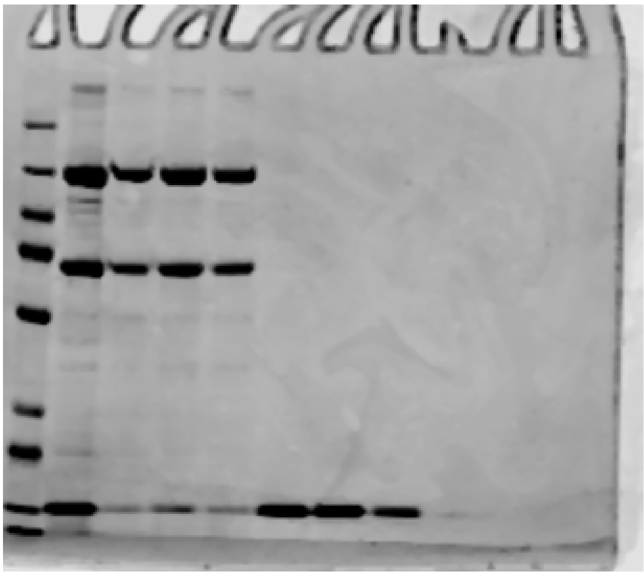

Supplement: Figure 1—figure supplement 2—source data 1. [file elife-109462-fig1-figsupp2-data1.pdf]
