# Supplementary figures and images for: Kinesin-1 conformational dynamics are controlled by a cargo-sensitive TPR switch
Source: eLife. 2026 Apr 14;14:RP109462. doi: 10.7554/eLife.109462 (PMC13078783; doi:10.7554/eLife.109462)

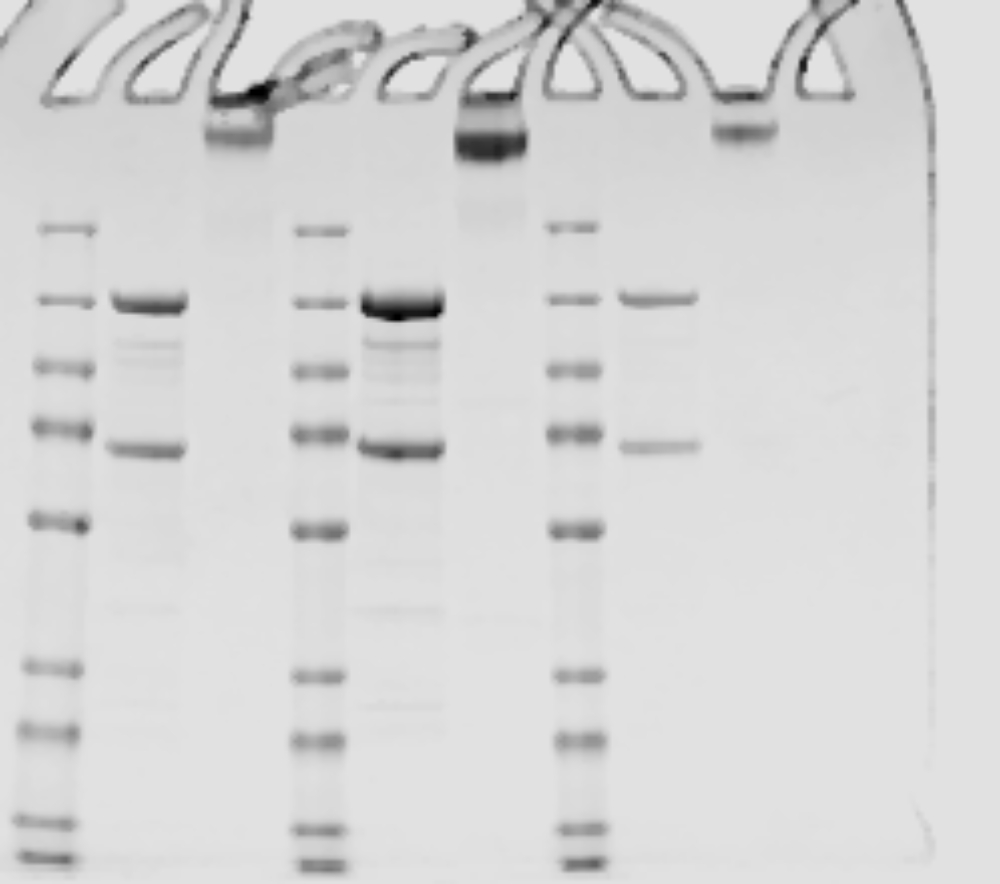

Supplement: Figure 1—figure supplement 2—source data 2. [file elife-109462-fig1-figsupp2-data2.zip › Figure 1-supplement 2-sourcedata 1.jpg]

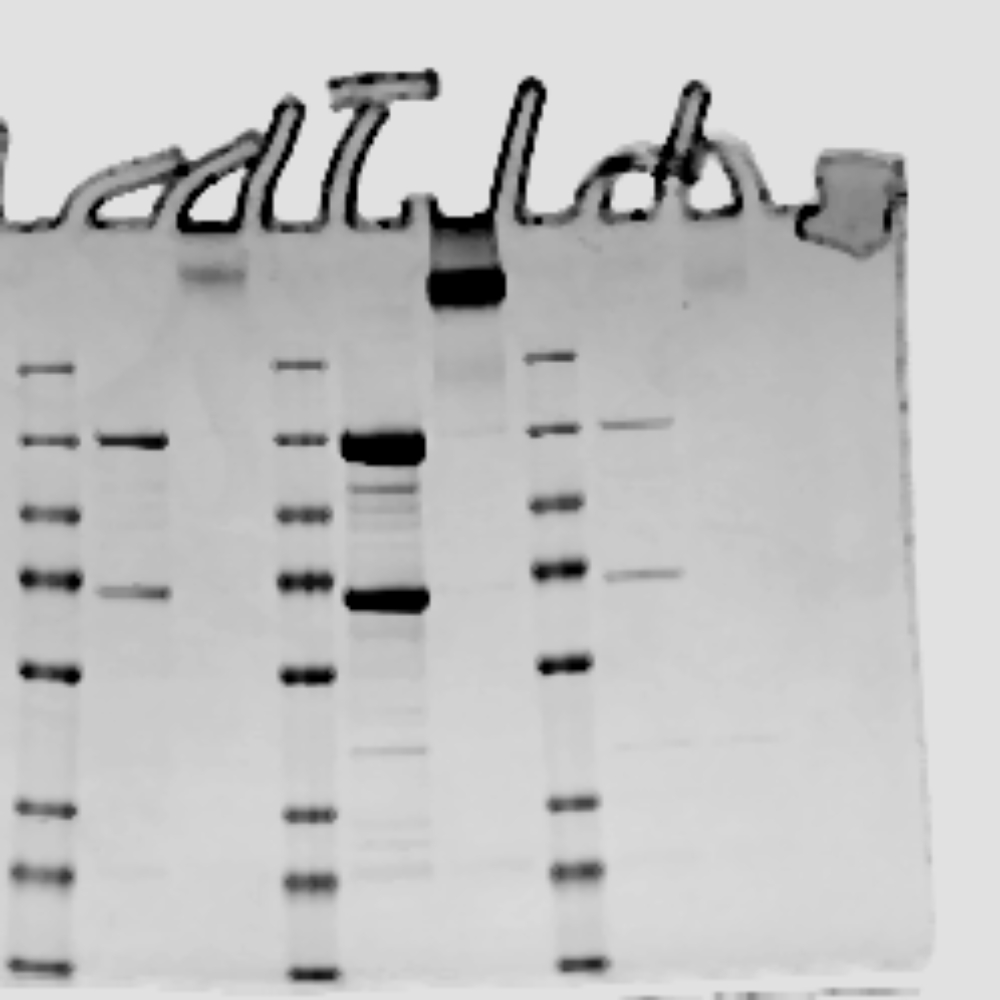

Supplement: Figure 1—figure supplement 2—source data 2. [file elife-109462-fig1-figsupp2-data2.zip › Figure 1-supplement 2-sourcedata 2.jpg]

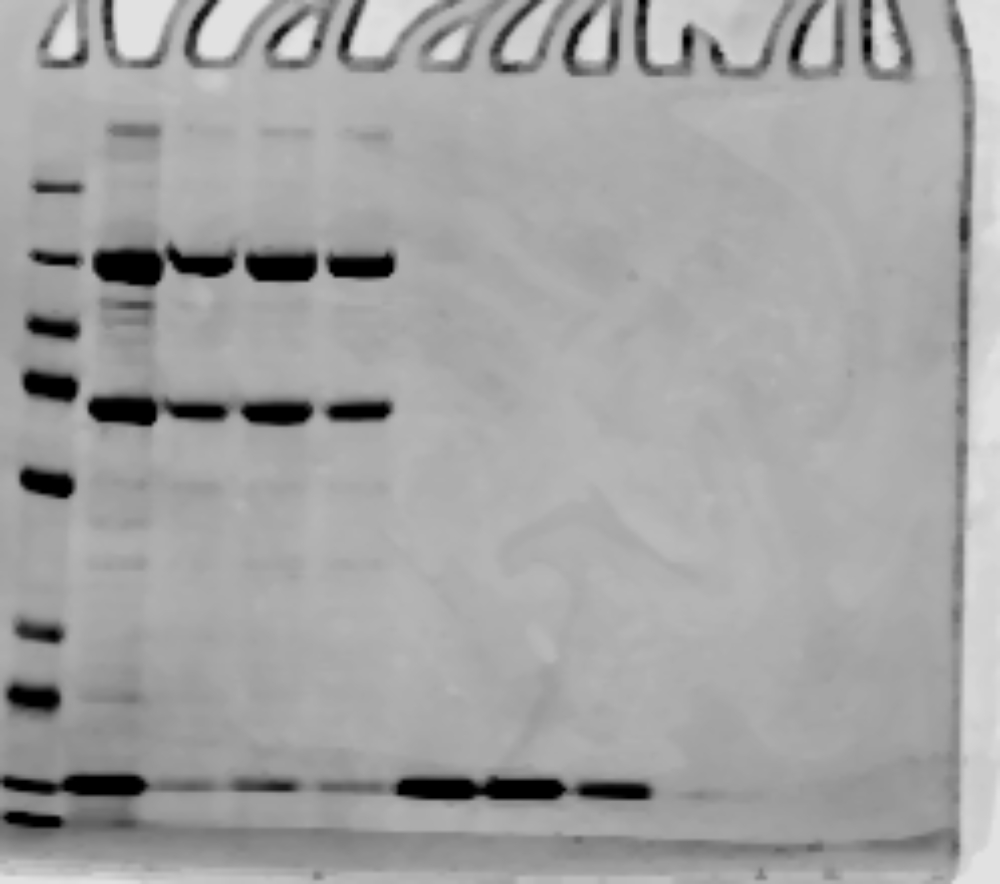

Supplement: Figure 1—figure supplement 2—source data 2. [file elife-109462-fig1-figsupp2-data2.zip › Figure 1-supplement 2-sourcedata 3.jpg]

Figure 1 -supplement 3

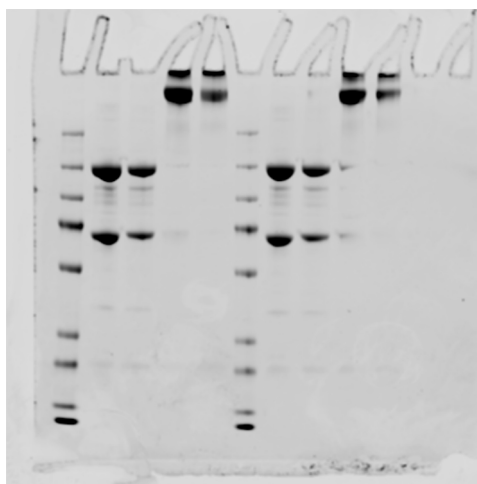

Supplement: Figure 1—figure supplement 3—source data 1. [file elife-109462-fig1-figsupp3-data1.pdf]

Input KHC / KLC uncropped

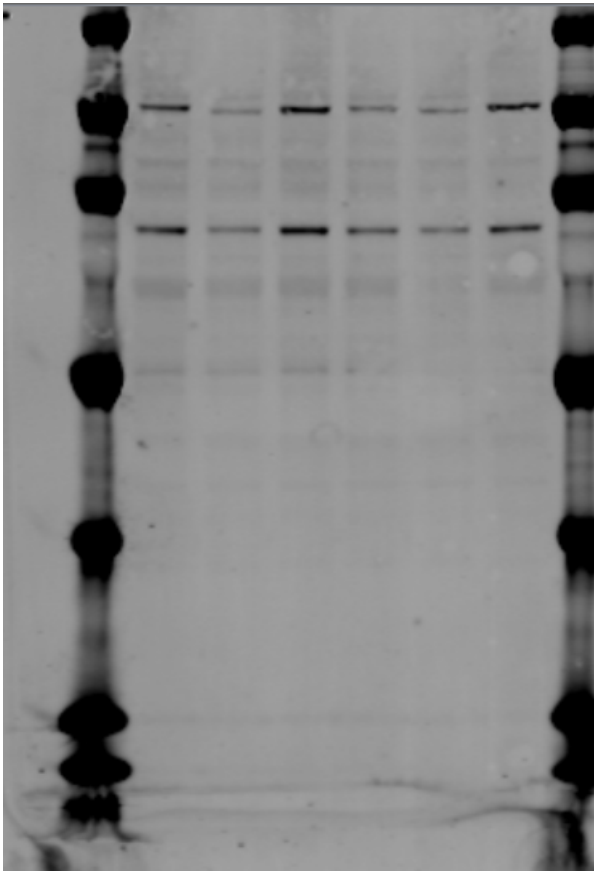

Bound KHC / KLC uncropped

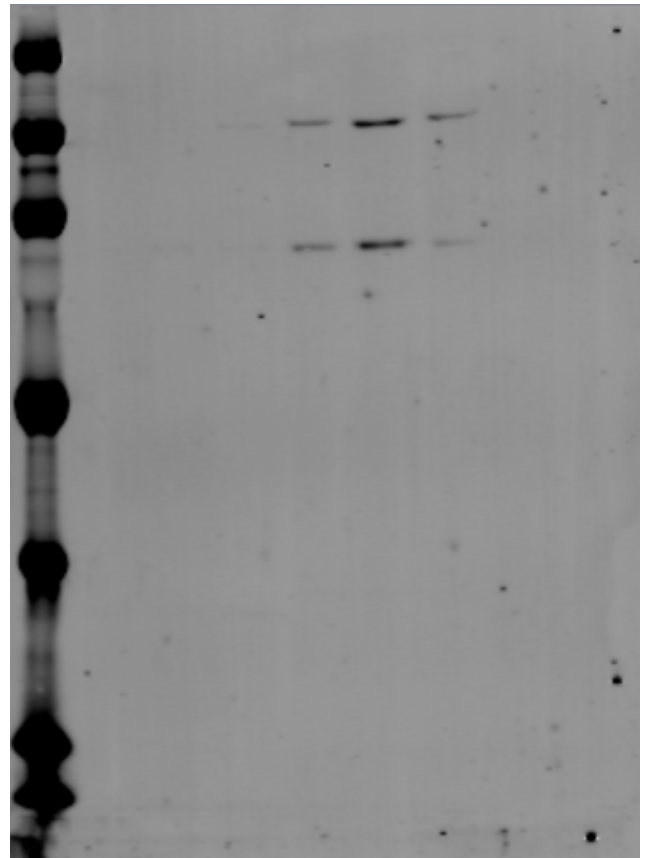

Supplement: Figure 5—source data 1. [file elife-109462-fig5-data1.pdf]

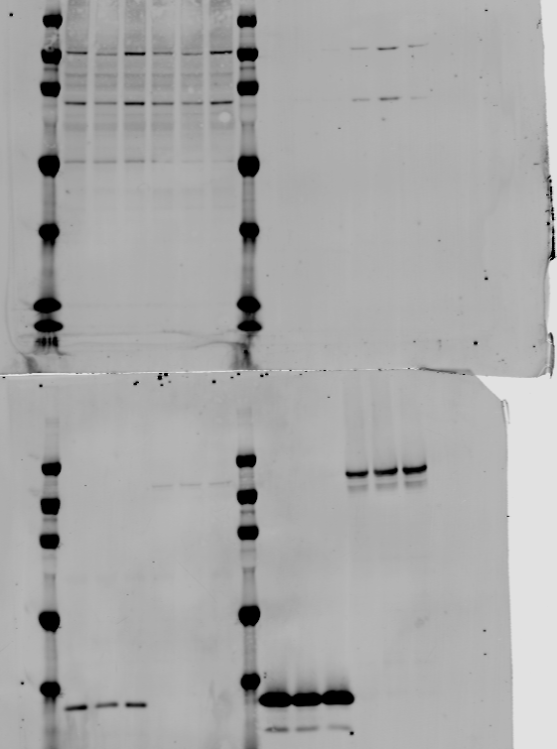

Supplement: Figure 5—source data 2. [file elife-109462-fig5-data2.zip › Figure5_Source_Data_2.tif]
