# Supplementary material for: Kinesin-1 conformational dynamics are controlled by a cargo-sensitive TPR switch
Source: eLife. 2026 Apr 14;14:RP109462. doi: 10.7554/eLife.109462 (PMC13078783; doi:10.7554/eLife.109462)
Supplement: Figure 4—source data 1. [file elife-109462-fig4-data1.docx]

| Data Set | Wild type; DeltaElbow; ElbowLock; ElbowLock-KinTag |
| --- | --- |
| HDX reaction details | 20mM Hepes, 150mM NaCl, 1mM MgCl2, 0.1mM ADP, 0.5mM TCEP, pD_read_ = 7.00, 23 °C |
| HDX time course (s) | 0.3, 0.5, 1, 3, 10, 30, 300 |
| Back-exchange (mean / IQR) |  |
| # of Peptides | 318 |
| Sequence coverage | 88% |
| Average peptide length / Redundancy | 14 / 3.55 |
| Replicates | 3 (technical) |
| Repeatability | 0.08 (average standard deviation) |
| Significant differences in HDX (delta HDX > X D) | 0.286 D (99% CI) |
